# Biomarker development for external CO_2_ injury prediction in apples through exploration of both transcriptome and DNA methylation changes

**DOI:** 10.1093/aobpla/plt021

**Published:** 2013-03-20

**Authors:** Nigel E. Gapper, David R. Rudell, James J. Giovannoni, Chris B. Watkins

**Affiliations:** 1 Department of Horticulture, Cornell University, Ithaca, NY 14853, USA; 2 Boyce Thompson Institute for Plant Research, Cornell University, Ithaca, NY 14853, USA; 3 Fruit Tree Research Laboratory, US Department of Agriculture/Agriculture Research Service, Wenatchee, WA 98801, USA; 4 Plant, Soil, and Nutrition Laboratory, US Department of Agriculture/Agriculture Research Service, Ithaca, NY 14853, USA

**Keywords:** Apple, biomarker, controlled atmosphere, DNA methylation, ethylene, external carbon dioxide injury, transcriptome

## Abstract

Several apple cultivars are susceptible to CO_2_ injury, a physiological disorder that can be expressed either externally or internally, and which can cause major losses of fruit during controlled atmosphere (CA) storage. Disorder development can also be enhanced using SmartFresh™ technology, based on the inhibition of ethylene perception by 1-methylcyclopropene (1-MCP). Injury development is associated with less mature fruit with lower ethylene production, but the aetiology of the disorder is poorly understood. Here we report on the progress made using mRNAseq approaches to explore the transcriptome during the development of external CO_2_ injury. Next-generation sequencing was used to mine the apple transcriptome for gene expression changes that are associated with the development of external CO_2_ injury. ‘Empire’ apples from a single orchard were treated with either 1 µL L^−1^ 1-MCP or 1 g L^−1^ diphenylamine or left untreated, and then stored in a CA of 5 kPa CO_2_ and 2 kPa O_2_. In addition, susceptibility to the disorder in the ‘Empire’ apples from five different orchards was investigated and the methylation state of the *ACS1* promoter investigated using McrBC endonuclease digestion and real-time quantitative polymerase chain reaction. Expression of over 30 000 genes, aligned to the apple genome, was monitored, with clear divergence of expression among treatments after 1 day of CA storage. Symptom development, internal ethylene concentrations (IECs) and methylation state of the *ACS1* promoter were different for each of five orchards. With transcriptomic changes affected by treatment, this dataset will be useful in discovering biomarkers that assess disorder susceptibility. An inverse correlation between the frequency of this disorder and the IEC was detected in a multiple orchard trial. Differential methylation state of the *ACS1* promoter correlated with both IEC and injury occurrence, indicating epigenetic regulation of ethylene biosynthesis and possibly events leading to disorder development.

## Abstract (precis)

Apple is a unique horticultural crop that is available to consumers year round, though harvested just once annually. A year-long supply is reliant on current postharvest practices such as refrigeration, controlled atmosphere and chemical treatment. However, disorders can develop during storage leading to loss of the crop at great cost to orchardists and storage facilities. The goal of this work is to develop predictive biomarkers the apple industry can use to market apples susceptible to disorders early, consequently reducing postharvest losses. This article outlines the genomics based approach we are taking to develop such tools, and presents our first list of putative predictive biomarkers.

## Introduction

Carbon dioxide injury is a physiological storage disorder that can occur during controlled atmosphere (CA) storage of several apple (*Malus sylvestris*) cultivars (Fidler *et al*. 1973; Fernandez-Trujillo *et al*. 2001). Susceptible cultivars include ‘Braeburn’, ‘Fuji’, ‘McIntosh’, ‘Cortland’ and ‘Empire’. The external form of the disorder is expressed as a bronze epidermal discolouration that is characterized by roughening of the skin, often partly sunken, with sharply defined edges (Blanpied *et al*. 1990). In a histological study of this disorder, Watkins *et al*. (1997) observed radially flattened and distorted hypodermal and outer cortex cells, in transverse sections of severely affected tissues, indicating cell collapse in these cell types. Within cultivars, susceptibility of fruit to injury can be orchard specific (Watkins *et al*. 1997), indicating an environmental factor in disorder risk.

Susceptibility of fruit to CO_2_ injury is at its highest during early CA storage, and the risk of injury can be reduced by maintaining low levels of CO_2_ in storage atmospheres during this early period of storage (Smock and Blanpied 1972; Watkins *et al*. 1997; Elgar *et al*. 1998; Argenta *et al*. 2000; Fawbush *et al*. 2008). Delaying the application of CA storage after harvest can also reduce injury (Watkins *et al*. 1997; Elgar *et al*. 1998; Colgan *et al*. 1999; Argenta *et al*. 2000; Fawbush *et al*. 2008). Application of diphenylamine (DPA), an antioxidant used commercially to control superficial scald (Lurie and Watkins 2012), also helps control CO_2_ injury (Burmeister and Dilley 1995; Watkins *et al*. 1997; Fawbush *et al*. 2008).

1-Methylcyclopropene (1-MCP), a potent inhibitor of ethylene action that interacts with and blocks ethylene receptors (Sisler and Serek 1997, 2003), is routinely used commercially on apples worldwide to maintain firmness and other quality attributes (Watkins 2008). However, 1-MCP treatment can cause a higher incidence of CO_2_ injury (DeEll *et al*. 2003; Watkins and Nock, 2004; Fawbush *et al*. 2008). The mechanism of 1-MCP action on CO_2_ injury is not known, but it prevents the reduction of injury associated with delayed CA (Fawbush *et al*. 2008; Argenta *et al*. 2010).

The objective of this study was to examine the molecular events leading to external CO_2_ injury in ‘Empire’ apples with the goal to developing biomarkers to predict susceptibility to this injury. We treated apples with both DPA and 1-MCP before CA storage, and monitored the occurrence of the disorder following treatment. The expression of over 30 000 genes was monitored in tissues collected from this experiment using mRNAseq transcriptome profiling. In addition, it has been noted that the environment has a major influence on the development of CO_2_ injury; susceptibility to this disorder varies within the same cultivar, from one orchard to another (Watkins and Liu 2010). DNA methylation has been observed as a control mechanism for biotic stress response (Dowen *et al*. 2012) and ripening (Zhong *et al*. 2013), so we carried out a pilot experiment to observe any evidence of differential DNA methylation in five different orchards at harvest.

## Methods

### Fruit

The experiments described were carried out over two harvest seasons. Fruit used in the first experiment were harvested from mature ‘Empire’ trees grown in a commercial orchard in the fall of 2010. This orchard block was chosen because of a previous history of fruit susceptibility to external CO_2_ injury. These fruit were treated with or without DPA or 1-MCP as detailed below. Apples were randomized and 40–45 fruit were used per treatment. Fruit used in Experiment 2 were harvested from five different orchard blocks in New York in the fall of 2011. These orchards were chosen because of a previous history of fruit susceptibility to external CO_2_ injury, with the aim of having some variation of the disorder represented. Apples were randomized, 20 fruit were used per treatment, and 40 fruit were visually assessed.

### 1-MCP, DPA and CA treatments

Fruit were treated for 24 h with 1 μL L^−1^ 1-MCP using SmartFresh™ tablets (AgroFresh Inc., Rohm & Haas Company, Spring House, PA, USA) in 4000-L plastic tents using a release and fan system supplied by the manufacturer. Fruit were dipped in 1 g L^−1^ DPA (Pace International, Wenatchee, WA, USA) for 1 min.

For Experiment 1, a CA of 5 kPa CO_2_ and 2 kPa O_2_ was applied to groups of 40–45 apples, each placed in a 19-L jar in a flow-through system as described by Fawbush *et al*. (2008), at 0.5 °C. For Experiment 2, a CA of 3 kPa CO_2_ and 2 kPa O_2_ was applied to groups of 20–40 apples placed in plastic pails, sealed in large CA chambers as described by Watkins and Nock (2012) at 0.5 °C. Peel tissue was sampled from the non-blushed side of fruit, snap frozen in liquid N_2_ and stored at −80 °C until used for further analyses.

### Visual assessment of injury

External CO_2_ injury was visually assessed on fruit removed from storage after 21 days after keeping fruit at 20 °C for 7 days.

### Internal ethylene concentration measurements

Internal ethylene concentration (IEC) measurements were carried out as described by Fawbush *et al*. (2008).

### RNA isolation and mRNAseq library construction

Total RNA was isolated from frozen, ground, apple peel tissue (300 mg) in 800 μL of the extraction buffer described by Chang *et al*. (1993), followed by the addition of 80 μL of 20 % sarkosyl. This mixture was vortexed and incubated at 65 °C for 10 min with occasional mixing by inversion. Chloroform (800 μL) was added and mixed by vortexing, and the mixture centrifuged at top speed in a bench-top centrifuge for 10 min at room temperature. The resulting aqueous phase was mixed with a half volume of ethanol, and then loaded and washed through a column following the manufacturer's instructions (RNAeasy, Qiagen). The highly purified total RNA was then treated with DNase I (Promega) at 37 °C for 30 min, followed by heat inactivation at 65 °C for 15 min. mRNAseq libraries were made using 2 μg of DNase-treated total RNA following the method of Zhong *et al*. (2011). In short, mRNA was isolated from total RNA, fragmented and used as a template to produce cDNA by reverse transcription using Superscript III (Invitrogen). Following first-strand cDNA synthesis, the second strand was synthesized with a dNTP mix incorporating dUTP instead of dTTP by DNA polymerase (Enzymatics). The ends of the double-stranded cDNAs were then repaired (Enzymatics), dA tailed by the Klenow enzyme (Enzymatics) and specific adapters ligated containing barcodes. Following the ligation of adapters, the second strand was digested by uracil DNA glycosylase (UDG) to enable strand-specific enrichment of the library. The UDG-digested cDNA was then used as a template to enrich the libraries by polymerase chain reaction (PCR) using the high-fidelity enzyme Phusion (NEB) with the following conditions: 95 °C for 2 min; 15 cycles of 98 °C for 11 s, 65 °C for 30 s, 72 °C for 25 s; 72 °C for 2 min; 4 °C soak. Libraries were quantified and 20 ng of each pooled for sequencing. A total of five libraries were multiplexed per sequencing reaction using an Illumina Genome Analyser II next-generation sequencer at the Weill Medicine School Sequencing Facility (Cornell University, New York City, NY, USA). One single biological sample was sequenced for each time point and treatment.

### Bioinformatics and statistical analyses

Short (40 bp) single-end, strand-specific RNA-Seq reads were filtered by aligning to adapter, rRNA and tRNA sequences using Bowtie (allowing two mismatches). The resulting high-quality reads were aligned to an apple predicted cDNA list (Velasco *et al.* 2010) using Tophat (allowing one segment mismatch) (Benjamini and Hochberg 1995). Following alignments, raw counts were normalized to reads per kilobase of exon model per million mapped reads. Reads that aligned to multiple predicted genes were discarded. Approximately 65 % of the sequence reads were able to be aligned to a predicted gene set. Aligned sequence reads ranged from 1.79 to 3.27 million reads per sample. Partial least square discriminant analysis was carried out using Unscrambler software (Camo, NJ, USA).

### Genomic DNA isolation, digestion and real-time quantitative PCR

Genomic DNA was extracted from apple peel tissue using a method based on that reported by Bernatzky and Tanksley (1986). The methylation state of the 2-kb promoter of *ACS1* was analysed based on the method of Holemon *et al.* (2007). Genomic DNA was digested using McrBC, an endonuclease that cleaves DNA containing methylcytosine on one or both strands, as per the manufacturer's instructions (New England Biolabs, NE, USA). Non-digested (control) and digested DNA samples were then subjected to real-time quantitative PCR (qPCR) using SYBR^®^ Green PCR Master Mix, and an ABI Prism 7900HT sequence detection system (Applied Biosystems, Foster City, CA, USA) with the primers designed to target the *ACS1* promoter listed in Table 1. The cycle threshold values of each sample were quantified using a standard curve generated for each primer pair, and non-digested DNA (control) was normalized to digested DNA.

**Table 1:** Primer sequences used for the DNA methylation study of the *ACS1* promoter.

Promoter region	Forward sequence	Reverse sequence
ACSpro1	5′-TCATTGCTTTGGGATTTGGGAGTGT-3′	5′-ATTGTACGCGCTTTTGCTCCACCA-3′
ACSpro2	5′-CGAGGCTCCAAGACAAAGAAATGGG-3′	5′-GGTGCTTGGTGGTTCTTCACAAGT-3′
ACSpro3	5′-TCGTACCGGATTTTCGAGGTTGAC-3′	5′-TCGCACAGGTTTTTATGTCTGCTCA-3′
ACSpro4	5′-ATATGACTACTCAGTGTGACGTGTC-3′	5′-GTGGGATATTGGATGTTGTTGGACT-3′
ACSpro5	5′-TGGATCATAATGTTAAGAAAAAGAACCAG-3′	5′-GTACCAAAAACATGATAAATAAATTATGAA-3′
ACSpro7	5′-TAAACTTTAACGCAAAACTCTCGGTAC-3′	5′-GGGTAAAGTGAATAGTAACAAGATTGA-3′

## Results

### Injury

In Experiment 1, 65 % of untreated fruit developed external CO_2_ injury, while 100 % of 1-MCP-treated fruit developed injury. Treatment of fruit with DPA reduced injury development to 2 %. In Experiment 2, injury averaged 70, 60, 84, 15 and 57 % in fruit from orchard blocks 1, 2, 3, 4 and 5, respectively. No significant differences were detected between untreated and 1-MCP-treated fruit (data not shown).

### mRNAseq results

**Figure 1.**
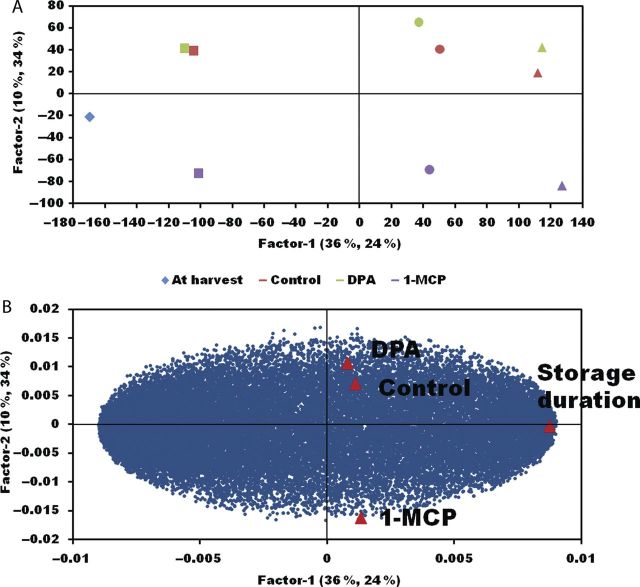
Partial least squares discriminant analysis of the ‘Empire’ transcriptome during aetiology of external CO_2_ injury. (A) Divergence of DPA and 1-MCP transcriptomes from the control. Closed diamond represents initial stage at harvest; closed squares represent 1 day of CA storage; closed circles represent 7 days of CA storage; closed triangles represent 14 days of CA storage. (B) Influence of storage duration, DPA and 1-MCP on the ‘Empire’ transcriptome.

A partial least squares discriminant analysis (PLSDA) was carried out using duration (storage time), DPA and 1-MCP treatment as *y* variables (Fig. 1). Duration had the greatest effect on the PLSDA model, while both DPA and 1-MCP treatments also had an effect. Within 1 day of storage, 1-MCP treatment caused transcriptomic divergence from untreated control fruit, indicating early expression changes in the apple transcriptome provoked by this treatment. By 7 days of storage, transcriptomic divergence from the control was observed for both 1-MCP and DPA treatments. Treatment with 1-MCP caused greater transcriptomic divergence from the control than DPA treatment.

**Figure 2.**
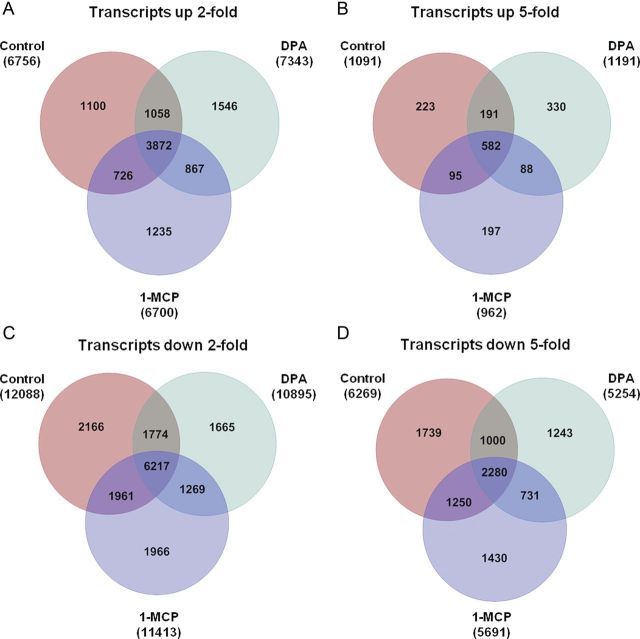
Changes in the transcriptome after 7 days of storage. (A) Transcripts up-regulated 2-fold after 7 days of storage compared with the initial stage at harvest. (B) Transcripts up-regulated 5-fold after 7 days of storage compared with the initial stage at harvest. (C) Transcripts down-regulated 2-fold after 7 days of storage compared with the initial stage at harvest. (D) Transcripts down-regulated 5-fold after 7 days of storage compared with the initial stage at harvest.

Transcriptome changes within the first 7 days of CA storage are shown in Fig. 2. For the 34 460 transcripts analysed, the abundance of ∼20 % increased 2-fold for control, DPA and 1-MCP treatments (Fig. 2A). Expression of ∼10 % of the transcriptome was elevated 2-fold for all treatments. Expression of ∼3 % of the transcriptome was elevated 5-fold for each of the three treatments and half of these transcripts were elevated for all treatments (Fig. 2B). More transcripts were down-regulated than up-regulated for all treatments. Between 32 and 35 % of the transcriptome was repressed 2-fold for each treatment, while ∼18 % of transcripts were repressed 2-fold for all treatments combined (Fig. 2C). Five-fold repression resulted in between 15 and 18 % of the transcriptome being reduced for each individual treatment, while half of these genes were repressed for all treatments combined (Fig. 2D).

**Figure 3.**
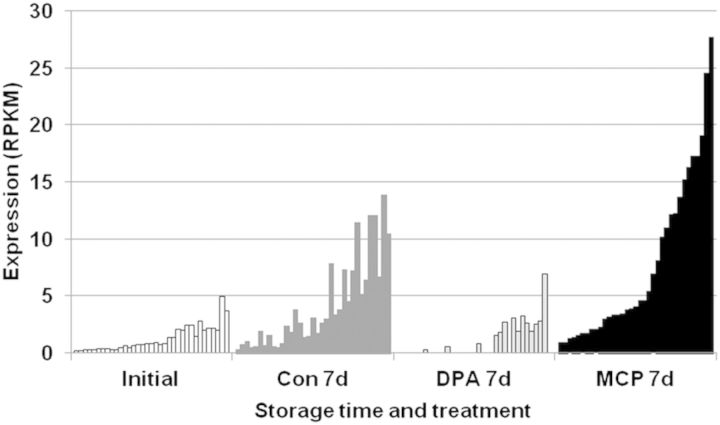
Differential abundance of putative biomarkers. Transcripts were selected, after 7 days of storage, based on the criteria that they were up-regulated 5-fold for 1-MCP and 2-fold for the control and were either repressed or unchanged for DPA, compared with the initial stage at harvest.

Transcripts were sorted to identify putative biomarkers within the first 7 days following storage inception. Expression profiles of 35 transcripts that were selected based on the criteria that they were up-regulated 5-fold for 1-MCP treatment and 2-fold for the control and were either repressed or unchanged for DPA are shown in Fig. 3. The biological function (GO terms) of these putative biomarkers is presented in the supplemental excel file **[Supporting Information]**.

### Methylation state of *ACS1* promoter

**Figure 4.**
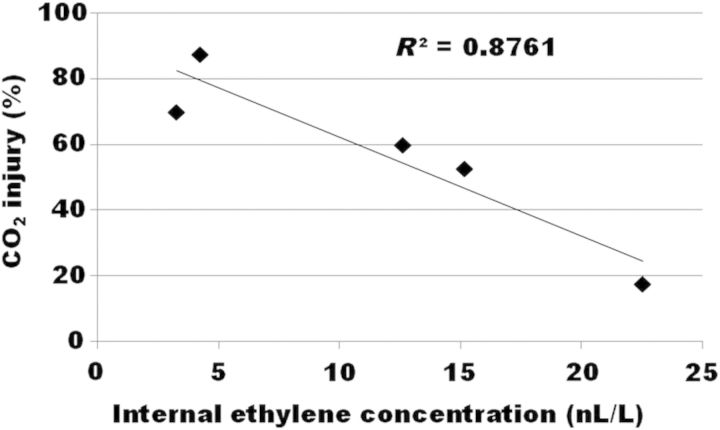
Relationship between external CO_2_ injury and IEC. The IECs and occurrence of injury following 21 days of CA storage and 7 days of shelf life storage are plotted.

**Figure 5.**
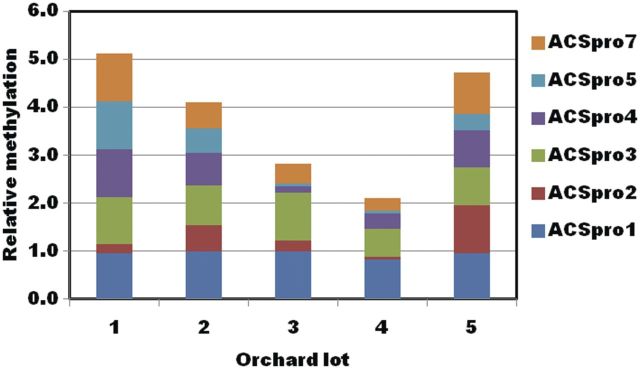
Differential methylation of the *ACS1* promoter. The relative methylation state of six different regions of the *ACS1* promoter were analysed from genomic DNA isolated from five different orchards at harvest. Genomic DNA was extracted from the peel of fruit and digested with an endonuclease that specifically digests methylated DNA. This DNA was then subjected to qPCR for each primer pair on the *ACS1* promoter, in both undigested and digested samples. The cycle threshold values of undigested DNA were normalized for digested DNA and presented as relative methylation.

**Figure 6.**
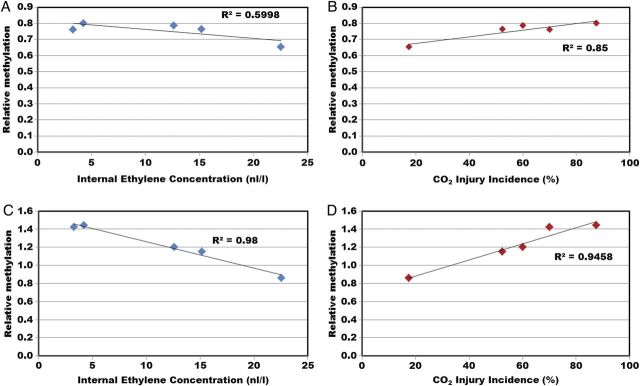
Relationship between DNA methylation of the *ACS1* promoter and IEC and external CO_2_ injury occurrence. (A) ACS1pro1 region, IEC. (B) ACS1pro1 region and injury. (C) ACS3pro1 region, IEC. (D) ACS1pro3 region and injury. Genomic DNA was extracted from the peel of fruit and digested with an endonuclease that specifically digests methylated DNA. This DNA was then subjected to qPCR for each primer pair on the *ACS1* promoter, in both undigested and digested samples. The cycle threshold values of undigested DNA were normalized for digested DNA and presented as relative methylation.

The relationship between external CO_2_ injury and IEC is shown in Fig. 4. An inverse correlation with an *R*^2^ value of 0.876 was observed. Primers were designed for the 2-kb promoter, 5′ upstream region of the *ACS1* gene. Genomic DNA was isolated from fruit before storage, and digested with McrBC, an endonuclease that targets methylated cytosine DNA sequences. Real-time quantitative PCR was performed and the resulting methylation states of each site of the *ACS1* promoter are shown in Fig. 5. Methylation varied considerably for each of the regions of the *ACS1* promoter chosen. The lowest methylation was found in orchard 4. The relationship of two regions of the *ACS1* promoter with IEC and injury, designated ACSpro1 and ACSpro3, was very strong (Fig. 6).

## Discussion

Post-harvest application of both DPA and 1-MCP was used to compare and contrast transcriptomes with the goal of identifying genes that regulate or mark the development of external CO_2_ disorder. Treatment with DPA almost eliminated the presence of the disorder in comparison with the control, while 1-MCP treatment enhanced disorder development compared with untreated control fruit. These findings are consistent with those of Fawbush *et al*. (2008).

### Transcriptome changes and putative biomarkers

Transcriptome changes were observed 1 day following CA storage inception for 1-MCP, and both DPA and 1-MCP treatments caused divergent changes in the transcriptome compared with untreated fruit (Fig. 1). However, the storage duration factor had the greatest influence on the PLSDA, indicating that the expression of more genes changed in response to storage duration than to treatment. Further, Fig. 2 illustrates the similarity of gene expression among treatments in the first 7 days of storage, both 2- and 5-fold up- or down-regulated, indicating a greater influence of storage duration than of treatments.

One longer-term goal is to identify gene transcripts that might be useful as biomarkers to predict risk of injury development. Transcripts were sorted using fairly stringent conditions during the first 7 days of storage: up-regulated 5-fold for 1-MCP treatment; up-regulated 2-fold in the control; and either repressed or not changed for DPA treatment. Only 35 genes met these criteria (Fig. 3). Not surprisingly, a few candidate predictive biomarkers have been identified from this analysis given the greater influence of storage duration on the transcriptome than of treatment. Nevertheless, some of these transcripts might be of use and require validation. In addition, the stringency of the filtering process can be lowered, and a comparison of other time points will also be helpful to capture more genes as putative biomarkers.

### Methylation of the *ACS1* promoter

Susceptibility of apples to external CO_2_ injury is normally enhanced by post-harvest treatments with 1-MCP, especially under high CO_2_ levels. This observation indicates that ethylene is likely to act in a mechanism of tolerance of the stress induced by both cold and elevated levels of CO_2_. It has also been noted, in the literature, that CO_2_ injury varies from orchard to orchard, indicating environmental control of the disorder. The inverse correlation of injury incidence and IEC in untreated fruit from the five orchards is an interesting finding. Environmentally induced impacts on injury might be related, in part, to epigenetic control of ethylene biosynthesis by a differential methylation state of genomic DNA. One candidate gene that would be the rate-limiting step of the ethylene biosynthetic pathway is *ACS1*. Zhong *et al*. (2013) observed methylation changes in the *ACS2* promoter in tomato during fruit development and ripening, so we decided to observe any methylation changes in the *ACS1* promoter on an orchard-to-orchard basis. Methylation of the *ACS1* promoter was different for each of the orchards (Fig. 5). Further, methylation states at two sites in the *ACS1* promoter region, ACSpro1 and ACSpro3, correlated very strongly with both IEC and injury (Fig. 6). Taken together, these results indicate plausible epigenetic control of *ACS1* transcript levels in these orchards, and it is likely that the expression of many other genes is also under the control of such mechanisms. Unfortunately, tissue was not harvested from the apples analysed for ethylene at 21 + 7 days of storage, so no direct comparison of ethylene and *ACS1* transcript levels is possible from these experiments. While still speculative, these findings are interesting, and further research is in progress.

## Conclusions

In addition to having an important biological role in the development of external CO_2_ injury, an epigenetically regulated transcript could provide a predictive marker that can be used by the apple industry. The aetiology of this disorder is rapid, which means that a useful predictive biomarker is probably required at harvest, or within the first few days of storage. Further studies are in place to capture the transcriptomes and methylomes of fruit in an orchard-to-orchard manner. A larger collection of tissue will be required, representing more orchards, to more clearly observe whether the environmental impact on this disorder can be attributed to an epigenetically regulated process, such as DNA methylation, and to mine for suitable epigenetically regulated predictive biomarkers. Certainly, an ‘at harvest’ biomarker, which is epigenetically regulated, would be the most easy to evaluate and would be potentially most useful as an industry tool.

## Sources of Funding

This research was supported by the USDA Specialty Crops Research Initiative (project number 2010-51181-21446).

## Contributions by the Authors

All the authors contributed to a similar extent overall to this work.

## Conflicts of Interest Statement

None declared.

## Supporting Information

The following additional information is available in the online version of this article –

**Table 1**. Gene Ontogeny (GO) terms of the 35 transcripts selected as putative biomarkers.

Additional Information
