# Supplementary material for: Biomarker development for external CO2 injury prediction in apples through exploration of both transcriptome and DNA methylation changes
Source: AoB Plants. 2013 Mar 20;5:plt021. doi: 10.1093/aobpla/plt021 (PMC3650494; doi:10.1093/aobpla/plt021)
Supplement: Additional Information [file supp_5_plt021_index.html]

Biomarker development for external CO2 injury prediction in apples through exploration of both transcriptome and DNA methylation changes — Biomarker development for external CO2 injury prediction in apples through exploration of both transcriptome and DNA methylation changes — Additional Information 

# Biomarker development for external CO2 injury prediction in apples through exploration of both transcriptome and DNA methylation changes

## Additional Information

**Files in this Data Supplement:**

- Additional Information - Additional Information
